# Supplementary material for: Feasibility of low-field magnetic resonance imaging (lf-MRI) for longitudinally evaluating experimentally induced lumbar intervertebral disc injuries in goat models (Capra hircus): A pilot study
Source: PLoS One. 2026 Feb 17;21(2):e0325577. doi: 10.1371/journal.pone.0325577 (PMC12912563; doi:10.1371/journal.pone.0325577)
Supplement: S1 Appendix — (DOCX) [file pone.0325577.s001.docx]

**Supplement 1: Standardized Protocol for Goat Lumbar IVD LFMRI Scanning**

1. Prepare scan parameters (Table 1) on MRI operator workstation
   1. Select 2D Spin Echo Thoracolumbar Spine Sequence optimized and saved for research study

Table 1: Sequence scan parameters

| **Parameter** | **Sequence name** | | |
| --- | --- | --- | --- |
|  | **PD Transverse** | **PD Sagittal** | **PD Dorsal** |
| Slice thickness (mm) | 5.5 | 5.5 | 5.5 |
| Slice spacing (mm) | 6 | 6 | 6 |
| Repetition time (TR [ms]) | 2150 | 1150 | 850 |
| Echo time (TE [ms]) | 26 | 26 | 26 |
| Number of averages (NEX) | 1 | 2 | 2 |

- 1. Enter new study info:
     1. Patient name: _______ [PI name and IACUC number]
     2. Animal ID: Goat _______[ear tag number of animal]
     3. Date:
     4. Sex:
     5. Species: Caprine
     6. Birth date:
     7. Description: thoracolumbar spine
     8. Owner name: PI
     9. Text box: name of study [ex. IVDD Pilot Study]

1. Prepare scanning room
   1. Ensure all personnel have removed ferromagnetic objects before entering scanner room
   2. Remove large table pad and replace with smaller moveable pads
   3. Stock room with plenty of towels and wedge sponges for placement or stabilizing areas
      1. *optional* preheat towels by placing in dryer briefly to help stabilize core body temperature
2. Prepare water phantoms
   1. Obtain two TB syringes and fill with distilled water.
   2. Arrange three strips (~8in in length) of tape parallel to each other with slight overlapping
   3. Place the two syringes on tape in alternating facing directions
   4. Place tape perpendicular to setup to secure syringes in place
   5. Obtain one long strip of tape (~24in in length or longer) and place parallel to the syringes centered.
3. Anesthetize animal as per attending veterinarian.
4. Transfer animal to MRI table
   1. Raise table to slightly higher than the MRI table
   2. Slide animal onto the pads in right lateral recumbency (Head facing MRI control room)
      1. *Ensure head is always supported and tubing cannot dislodge*
   3. Once animal is slid generally into the gantry field ensure all sensors (EKG, pulse ox, etc) are placed and reading
   4. Secure sensors
   5. Slide animal further into the gantry
      1. Last rib should be on the within the field of view
      2. Due to the ribs being hard to trace in the narrow space the top of the rib over the abdominal cavity should be approximately 3-4in from the left edge of the gantry
5. Refine animal positioning to ensure lumbar spine is centered and completely included in the scan field of view
   1. Once ribs are generally in place, reach the long tape through and use the spinal processes as a guide for placing the syringes. At least one syringe must be over L1-L3.
   2. Elevate shoulders and head with wedge sponge to minimize rumen fluid leakage
   3. Anchor hind to rear cleat first to help pull animal into gantry
   4. Anchor front limbs to cleat closest to control room
   5. Insert rolled towels between forelimbs and hindlimbs to minimize lumbar spine rotational obliquity.
   6. Extend thoracolumbar spine as flat as possible and center cranial lumbar spine within the spine coil. Include the last pair of ribs.
6. Acquire localizer images
   1. adjust positioning as needed to ensure spine is in the isocenter for all 3 planes
7. Acquire scout sequence
   1. Pause for anesthesia check when done
   2. Ensure dorsal images include ribs and all margins of L1-5 IVDs
   3. Ensure sag images include all margins of L1-5 IVDs
   4. Ensure trans images include discs of interest and water phantom syringe
8. Acquire PD dorsal planar pulse sequence
   1. Check sagittal scout view and adjust slice plane as needed to ensure that slice plane is parallel to long axis of spine
   2. Check transverse scout view and adjust slice plane as needed to ensure slice plane is aligned with transverse processes
   3. Start aquisition
   4. pause for anesthesia check after acquisition done
   5. check image quality and ensure T13 ribs and L1-5 IVDs are well visualized
9. Acquire PD sagittal planar pulse sequence
   1. Check dorsal planar scout view and adjust slice plane as needed to ensure slice plane is parallel to long axis of spine
   2. Check transverse scout view and adjust slice plane as needed to ensure slice plane is aligned with dorsal spinous process of vertebra.
   3. Start acquisition
   4. pause for anesthesia check when done
   5. check image quality and ensure that L1-4 IVDs are well-visualized
10. Acquire PD transverse pulse sequence
    1. Check sagittal and dorsal planar scout views and adjust slice plane as needed to ensure slice plane is perpendicular to spinal canal and parallel to vertebral endplates
    2. Start acquisition
    3. Pause for anesthesia check when done
11. Review all scans to ensure they are of diagnostic quality with no motion artifacts
12. Remove animal from table and recover from anesthesia as per attending veterinarian
