# Supplementary material for: Feasibility of low-field magnetic resonance imaging (lf-MRI) for longitudinally evaluating experimentally induced lumbar intervertebral disc injuries in goat models (Capra hircus): A pilot study
Source: PLoS One. 2026 Feb 17;21(2):e0325577. doi: 10.1371/journal.pone.0325577 (PMC12912563; doi:10.1371/journal.pone.0325577)
Supplement: S2 Appendix — (DOCX) [file pone.0325577.s002.docx]

**Supplemental Appendix 2. Standardized Protocol for Goat Lumbar Disc lf-MRI Measurements**

1. Open Horos software application.
2. Select MRI patient study to be analyzed from database list and open.
3. Open the MRI study within this folder that is identified as “PD sagittal”.
4. Once opened, adjust the mouse functions for the left/right button and the roller
   - Left: Density adjustment
   - Right: Zoom
   - Roller: Reposition image
     - To utilize each of these functions, the button must be depressed before moving the mouse to make the adjustment


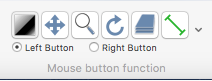


1. Set the WL/WW to the “default” setting using the drop down menu
2. In the toolbar, select “3D viewer”, then select “3D-MPR” from the dropdown menu.


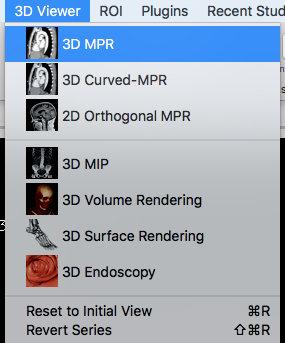


1. Left click on the sagittal planar image and use the image rotate tool to orient the patient’s head to the left and the tail to the right
2. Left click on the dorsal planar image and pan through the slices using the mouse roller until the last set of ribs becomes visible. Identify the disc space directly below them as “T13-L1” and use this landmark as the basis for identify the remaining lumbar discs.


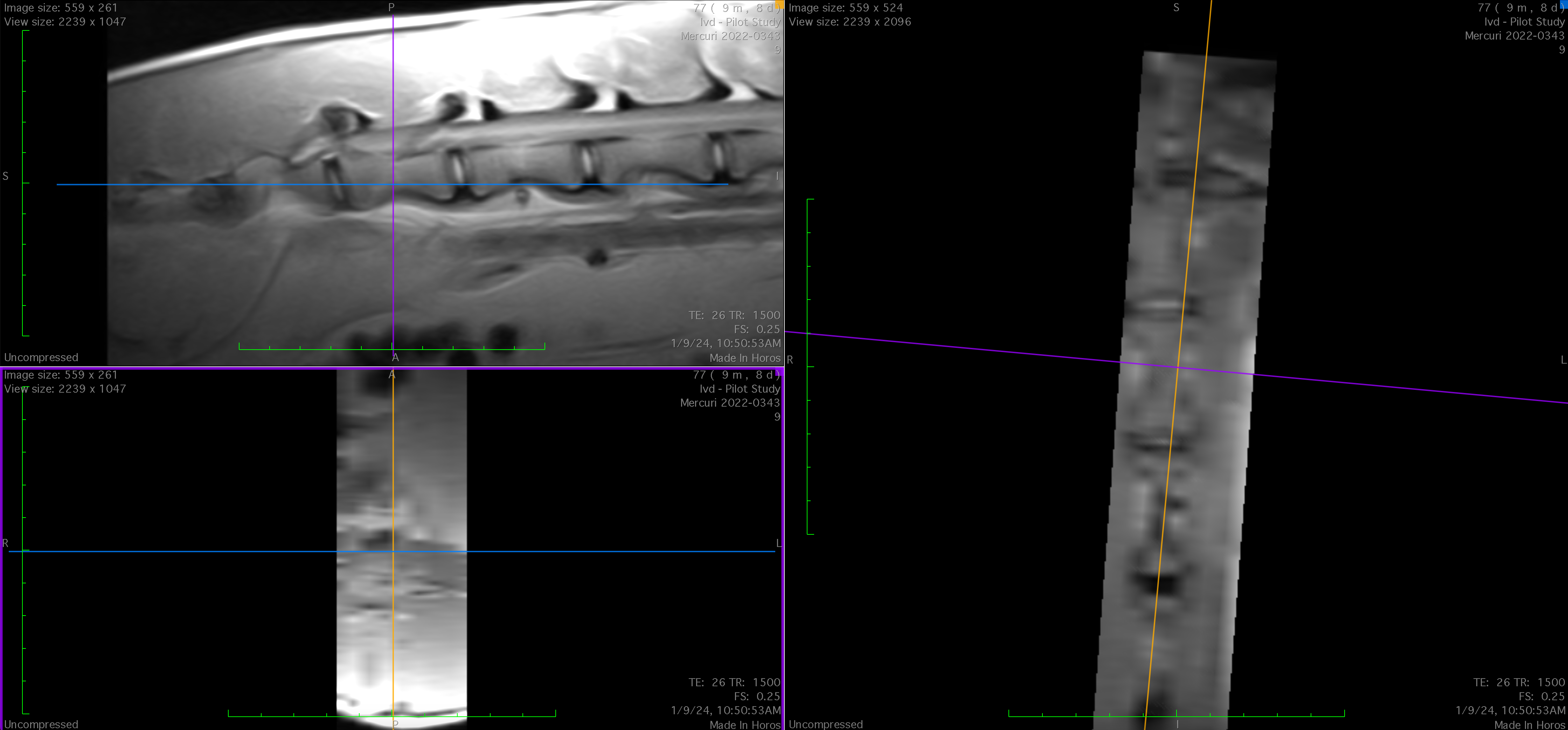


1. Left click the dorsal planar image and adjust the yellow line as needed so the line is in the center of the discs and parallel to the long axes of the vertebral bodies.


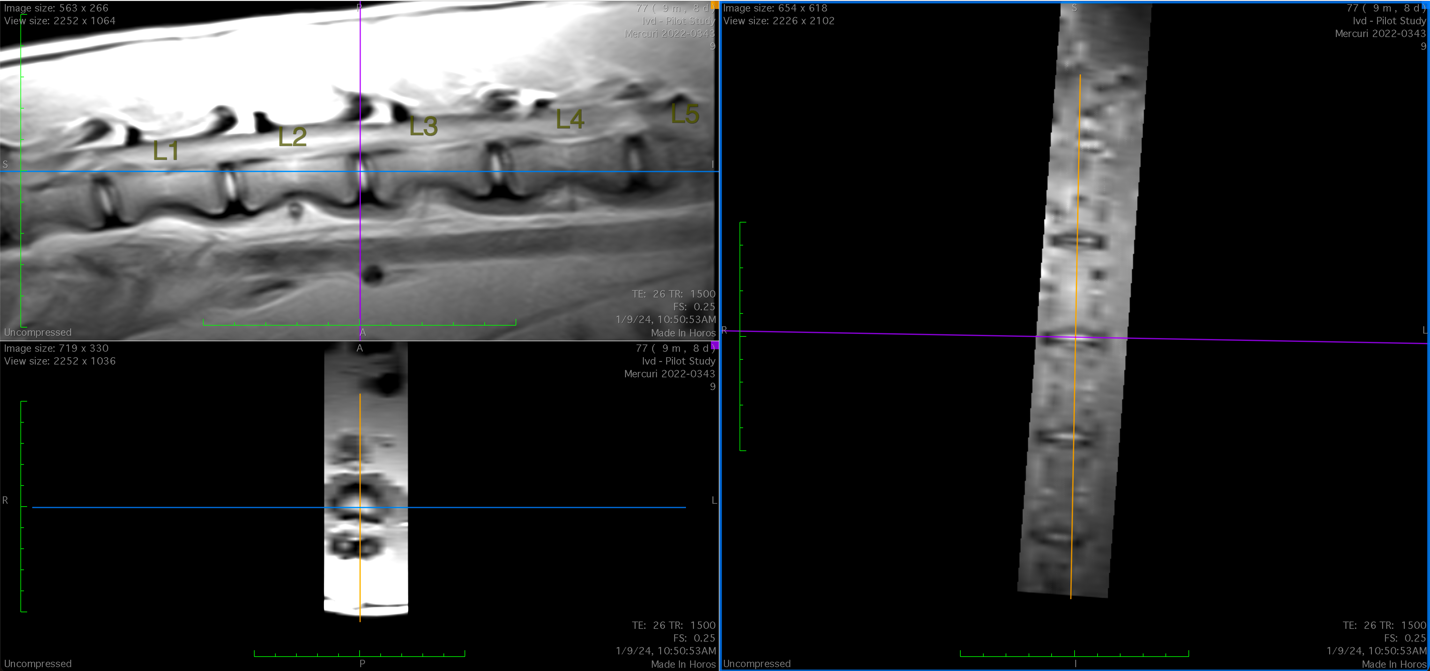


1. Double click on the sagittal view so it will fill the screen.
2. Measure the nucleus pulposus values for calculating MRI index
   1. Select the pencil tool by clicking the down arrow next to the green length measuring tool.


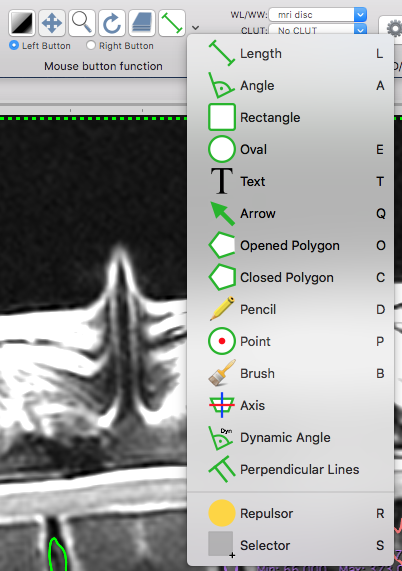


- 1. Hold down the left mouse button to trace the outer margins of the NP. The software will produce the area measurement, mean signal intensity, and standard deviation of signal intensity.
  2. Repeat this process for all lumbar discs on scan working back from T13-L1.
  3. Record each of values for the NP, signal intensity (mean), and standard deviation
  4. Use spreadsheet software to multiply the value of NP area and signal intensity to calculate the MRI index values.
  5. Delete the area tracings by selecting “ROI” from the top toolbar and “delete all ROIs in this series” from the pulldown.

1. Measure dorsal, middle, and ventral disc heights.
   1. Select the line tool from the top toolbar. Using the electronic cursors, place two lines at the dorsal and ventral extremes of the vertebrae cranial and caudal to the disc of interest such that each line starts at the caudal subchondral margin of one vertebral body and ends on the cranial subchondral margin of the adjacent vertebra. Record the length values as ventral and dorsal disc heights.


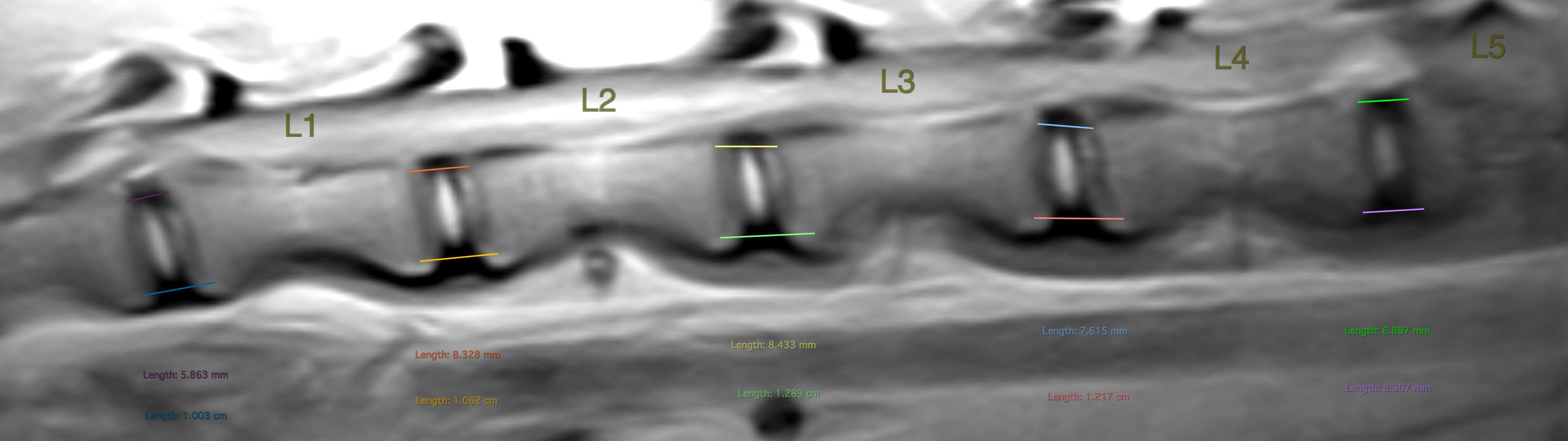


- 1. Place a third line perpendicular to these ventral and dorsal lines.
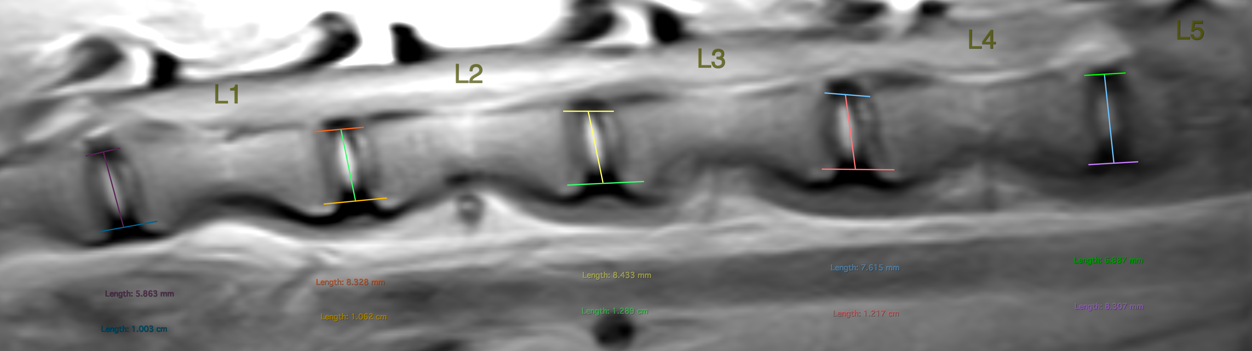

  2. Using a calculator, divide the length of the line made in step 12b.by 2 and place a fourth line parallel to this line starting at either the dorsal or ventral disc height line.


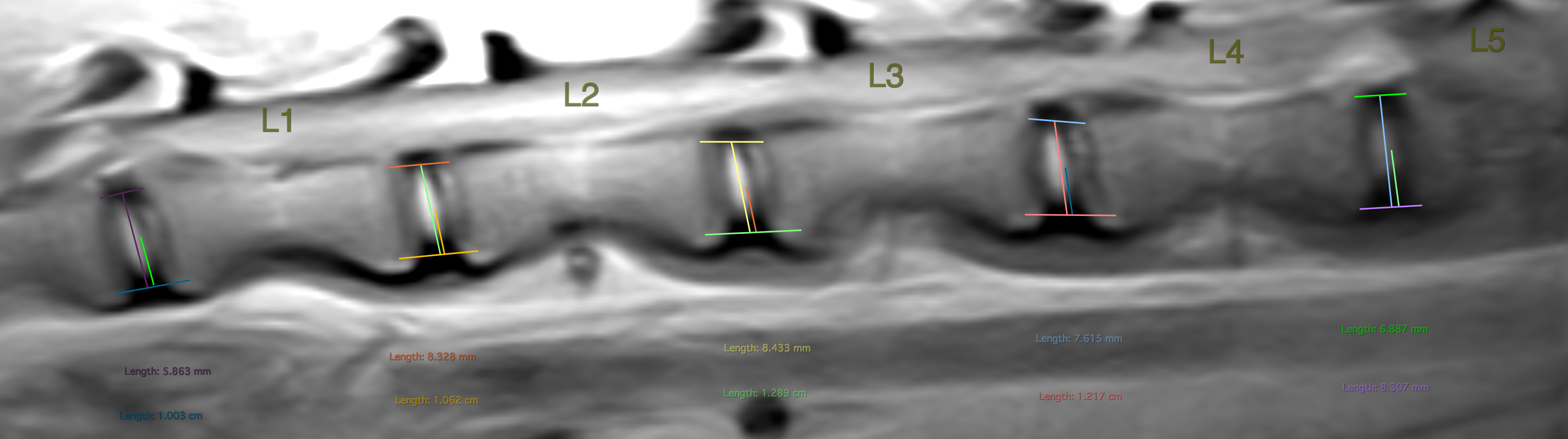


- 1. Create a 5^th^ line at this middle location of the disc and place cursors at the caudal subchondral margin of one vertebra and the cranial subchondral margin of the next vertebra. The line should be roughly parallel with the ventral disc height length and lie atop the endpoint of the line drawn in step 12c. for the respective disc. Record the length as middle disc height.


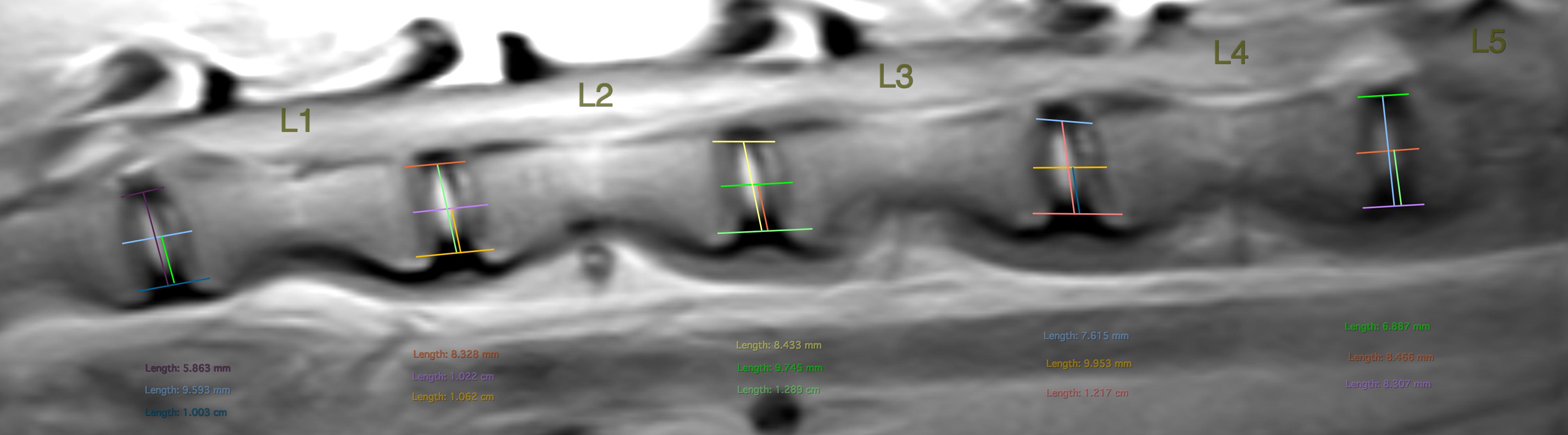


1. Place a 6^th^ line extending from the caudal endpoint of the line used for measuring the middle disc height to the cranial endpoint of the line indicating the middle disc height of the subsequent intervertebral disc. Record the length value as the cranial vertebral body height (cranial to the intervertebral disc directly to the right of it).


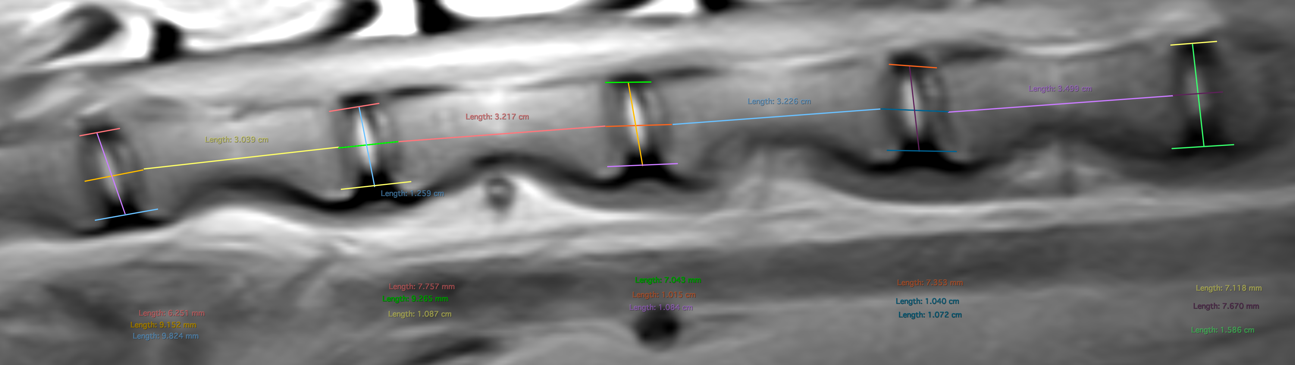


1. If margins of the NP or the vertebral subchondral bone are not distinguishable, record these values as “indeterminate” and exclude them from analyses (i.e. severe degeneration or inflammation).
