# Supplementary material for: Feasibility of low-field magnetic resonance imaging (lf-MRI) for longitudinally evaluating experimentally induced lumbar intervertebral disc injuries in goat models (Capra hircus): A pilot study
Source: PLoS One. 2026 Feb 17;21(2):e0325577. doi: 10.1371/journal.pone.0325577 (PMC12912563; doi:10.1371/journal.pone.0325577)
Supplement: S3 Appendix — (DOCX) [file pone.0325577.s003.docx]

**Supplemental Appendix 3.** Lf-MRI findings recorded by a veterinary radiologist immediately following image acquisition for each goat at each time point.

| **Time point** | **Goat 77** | **Goat 79** | **Goat 80** |
| --- | --- | --- | --- |
| Pre-operative | Normal | Normal | Normal |
| 3-weeks post-operative | - space-occupying, heterogenous, ill-defined tissue in paraspinal region and abdominal wall consistent with post-operative granulation tissue - enlargement of left hypaxial muscle group, with loss of margination and possible fluid in intermuscular space - discrete, scalloped defects in L1-2 endplate margins consistent with iatrogenic endplate injury or early discospondylitis | - heterogenous, ill-defined tissue in left paraspinal region and abdominal wall with fat stranding and some rounded areas of increased intensity consistent with fluid pockets - enlargement of intermuscular spaces in left hypaxial muscle group, with some areas of increased muscle signal intensity and blunting of margins - focal loss of margination in caudal T13 endplate margin visible in sagittal image only | - increased disc signal, loss of endplate margins, and increased subchondral signal at L2-3 and L3-4 consistent with iatrogenic disc and endplate injury or discospondylitis - increased left hypaxial muscle signal with blunted margins and widened intermuscular spaces consistent with iatrogenic muscle injury with edema - heterogenous soft tissue extending from paraspinal muscles to abdominal wall with some rounded hyperintense foci consistent with post-operative granulation tissue with seromas or early abscesses |
| 6 weeks post-operative | - increased disc signal, vertebral sclerosis, annulus protrusion, and endplate lysis at L1-2 and L2-3 consistent with discospondylitis - increased hypaxial muscle signal intensity with enlarged and irregular left hypaxial muscle from mid L1 to mid-L3 - oblong, intermediate signal structures on either side of aorta at mid L1 consistent with enlarged lymph nodes - heterogenous, proliferative tissue extending from left hypaxial muscle to abdominal wall consistent with post-operative granulomatous or pyogranulomatous tissue - subjectively small rumen consistent with decreased food ingestion | - subjective decrease in disc signal L2-3 consistent with early degeneration - heterogenous, tissue in left paraspinal region and abdominal wall with fat stranding consistent with granulation tissue - enlargement of intermuscular spaces in left hypaxial muscle group, with some areas of increased muscle signal intensity and blunting of margins consistent with muscle fibrosis - intermediate signal tissue in intervertebral foramina L2-3 and L3-4 consistent with disc margin protrusion and epidural fibrosis | - increased disc signal, increased disc space width, loss of endplate margins, and decreased vertebral body signal at l2-3 and l3-4 consistent with discospondylitis - increased left hypaxial muscle signal with blunted margins and widened intermuscular spaces consistent with muscle fibrosis - fat stranding in left paraspinal region consistent with post-operative granulation tissue |
| 12 weeks post-operative | - partial resolution of previous increased disc signal with progressive vertebral sclerosis, annulus protrusion, and proliferative, paravertebral tissue at L1-2 and L2-3 consistent with chronic discospondylitis with periosteal proliferation secondary to adjacent soft tissue inflammation - increased hypaxial muscle signal intensity with enlarged and irregular left hypaxial muscle from mid L1 to mid-L3 consistent with myositis - persistent oblong, intermediate signal structures on either side of aorta at mid L1 consistent with lymphadenopathy - partially resolved heterogenous, proliferative tissue extending from left hypaxial muscle to abdominal wall consistent with chronic granulomatous or pyogranulomatous tissue | - subjective decrease in disc signal and disc space width L2-3 consistent with early degeneration - partial resolution of heterogenous, tissue in left paraspinal region and abdominal wall with fat stranding consistent with resolving granulation tissue - enlargement of intermuscular spaces in left hypaxial muscle group, with some areas of increased muscle signal intensity and blunting of margins consistent with chronic postoperative muscle fibrosis - intermediate signal tissue in intervertebral foramina L2-3 and L3-4 consistent with disc margin protrusion, epidural fibrosis - articular process hypertrophy consistent with degenerative joint disease | - partial resolution of previous increased disc signal, increased disc space width, loss of endplate margins, decreased vertebral body signal at L2-3 and L3-4, enlarged medial iliac lymph node consistent with chronic discospondylitis - increased left hypaxial muscle signal with blunted margins and widened intermuscular spaces consistent with chronic post operative muscle fibrosis - fat stranding in left paraspinal region consistent with chronic resolving post-operative granulation tissue. - articular process hypertrophy with osteophytes consistent with degenerative joint disease |
