# Supplementary material for: Feasibility of low-field magnetic resonance imaging (lf-MRI) for longitudinally evaluating experimentally induced lumbar intervertebral disc injuries in goat models (Capra hircus): A pilot study
Source: PLoS One. 2026 Feb 17;21(2):e0325577. doi: 10.1371/journal.pone.0325577 (PMC12912563; doi:10.1371/journal.pone.0325577)
Supplement: S4 Appendix — (DOCX) [file pone.0325577.s004.docx]

**Supplemental Appendix 4:: DHI repeatability analysis details**

**DHI Intra-observer Repeatability**

**Day 0**

- For the bone-to-bone and endplate-to-endplate DHI of observer CH, 8.33% (1 of 12) and 25% (3 of 12) of CV measures were found to not be repeatable, respectively
- For the bone-to-bone and endplate-to-endplate DHI of observer MK, 58.3% (7 of 12) and 100% (12 of 12) of CV measures were found to not be repeatable, respectively

**Week 3**

- For the endplate-to-endplate DHI of observer CH, 14.29% (1 of 7) of CV measures were found to not be repeatable. All bone-to-bone DHI measures had acceptable repeatability.
- For the bone-to-bone and endplate-to-endplate DHI of observer MK, 28.57% (2 of 7) and 42.86% (3 of 7) of CV measures were found to not be repeatable, respectively.

**Week 6**

- For the bone-to-bone and endplate-to-endplate DHI of observer CH, 14.29% (1 of 7) and 42.86% (3 of 7) of CV measures were found to not be repeatable, respectively.
- For the bone-to-bone and endplate-to-endplate DHI of observer MK, 57.14% (4 of 7) and 14.29% (1 of 7) of CV measures were found to not be repeatable, respectively.

**Week 12**

- For the bone-to-bone DHI of observer CH, 12.5% (1 of 8) of CV measures were found to not be repeatable. All endplate-to-endplate DHI measures had acceptable repeatability.
- For the bone-to-bone DHI of observer MK, 25% (2 of 8) of CV measures were found to not be repeatable. All endplate-to-endplate DHI measures had acceptable repeatability.

**DHI Inter-observer Repeatability**

**Day 0**

- For the bone-to-bone disc height index (DHI), mean CVs for observers MK and CH were 10.077 and 4.858, respectively. The means were significantly different as indicated by the p-value of 0.0038.
- For the endplate-to-endplate DHI, mean CVs for observers MK and CH were 16.901 and 6.640, respectively. The means were significantly different as indicated by the p-value of <0.0001.

**Week 3**

- For goat 77, discs L1/2 and L2/3 were excluded from analyses. Discs L2/3 and L3/4 were excluded for goat 80. Exclusions were due to unclear margins attributed to an inflammatory response that inhibited the observers' ability to differentiate between anatomical structures needed to make measurements and reduced image quality attributed to relevant discs being near the end of the field of view. These exclusions were made for weeks 6 and 12 also.
- For the bone-to-bone DHI, mean CVs for observers MK and CH were 7.97 and 3.89, respectively. The means were significantly different as indicated by the p-value of 0.0188.
- For the endplate-to-endplate DHI, mean CVs for observers MK and CH were 11.475 and 8.212, respectively. The means were not significantly different (p = 0.3446).

**Week 6**

- For the bone-to-bone DHI, mean CVs for observers MK and CH were 6.737 and 3.397, respectively. The means were not significantly different (p=0.0998)
- For the endplate-to-endplate DHI, mean CVs for observers MK and CH were 5.703 and 7.036, respectively. The means were not significantly different (p = 0.5091).

**Week 12**

- For the bone-to-bone DHI, mean CVs for observers MK and CH were 7.581 and 5.136, respectively. The means were not significantly different (p = 0.2415).
- For the endplate-to-endplate DHI, mean CVs for observers MK and CH were 6.360and 4.200, respectively. The means were not significantly different (p = 0.1668).
