# Supplementary material for: Feasibility of low-field magnetic resonance imaging (lf-MRI) for longitudinally evaluating experimentally induced lumbar intervertebral disc injuries in goat models (Capra hircus): A pilot study
Source: PLoS One. 2026 Feb 17;21(2):e0325577. doi: 10.1371/journal.pone.0325577 (PMC12912563; doi:10.1371/journal.pone.0325577)
Supplement: S5 Appendix — Measurements were recorded in triplicate for all discs unless they were excluded from analyses due to non-infectious discospondylitis making margins of the nucleus pulposus and vertebral endplates indistinguishable. (DOCX) [file pone.0325577.s005.docx]

**Supplemental Appendix 5:** Disc Height Index Co-efficients of variation for each observer, time point, animal ID, disc level, and measurement method. Measurements were recorded in triplicate for all discs unless they were excluded from analyses due to an inflammatory response making margins indistinguishable.

| **Observer** | **Time Point (Weeks)** | **Animal ID** | **Disc Level** | **Number of replications** | **CV (Bone to Bone - DHI)** | **CV (Endplate to Endplate - DHI)** |
| --- | --- | --- | --- | --- | --- | --- |
| Observer 1 | 0 | 77 | L1/L2 | 3 | 1.857672058 | 2.191606526 |
| Observer 1 | 0 | 77 | L2/L3 | 3 | 6.876406835 | 5.502551504 |
| Observer 1 | 0 | 77 | L3/L4 | 3 | 1.753114661 | 2.199375035 |
| Observer 1 | 0 | 77 | L4/L5 | 3 | 5.620303469 | 6.367927048 |
| Observer 1 | 0 | 79 | L1/L2 | 3 | 3.124071361 | 7.366039114 |
| Observer 1 | 0 | 79 | L2/L3 | 3 | 1.716531897 | 10.2006818 |
| Observer 1 | 0 | 79 | L3/L4 | 3 | 8.105582912 | 12.89267993 |
| Observer 1 | 0 | 79 | L4/L5 | 3 | 0.895896146 | 15.64732402 |
| Observer 1 | 0 | 80 | L1/L2 | 3 | 4.365953305 | 7.208621518 |
| Observer 1 | 0 | 80 | L2/L3 | 3 | 9.259009016 | 0.93059862 |
| Observer 1 | 0 | 80 | L3/L4 | 3 | 2.907943782 | 4.575198388 |
| Observer 1 | 0 | 80 | L4/L5 | 3 | 11.81076519 | 4.599124555 |
| Observer 2 | 0 | 77 | L1/L2 | 3 | 13.98353902 | 12.75776773 |
| Observer 2 | 0 | 77 | L2/L3 | 3 | 5.953836004 | 16.31241519 |
| Observer 2 | 0 | 77 | L3/L4 | 3 | 12.17169669 | 14.18327103 |
| Observer 2 | 0 | 77 | L4/L5 | 3 | 17.20620877 | 22.17674417 |
| Observer 2 | 0 | 79 | L1/L2 | 3 | 1.06928594 | 14.10941393 |
| Observer 2 | 0 | 79 | L2/L3 | 3 | 8.862007008 | 19.17189244 |
| Observer 2 | 0 | 79 | L3/L4 | 3 | 11.94552195 | 26.90925378 |
| Observer 2 | 0 | 79 | L4/L5 | 3 | 6.309826415 | 23.34782885 |
| Observer 2 | 0 | 80 | L1/L2 | 3 | 12.2636769 | 11.37944726 |
| Observer 2 | 0 | 80 | L2/L3 | 3 | 11.49073156 | 13.08641093 |
| Observer 2 | 0 | 80 | L3/L4 | 3 | 8.762290535 | 14.48315765 |
| Observer 2 | 0 | 80 | L4/L5 | 3 | 10.90087087 | 14.89436754 |
| Observer 1 | 3 | 77 | L1/L2 | 0 |  |  |
| Observer 1 | 3 | 77 | L2/L3 | 0 |  |  |
| Observer 1 | 3 | 77 | L3/L4 | 3 | 4.412153289 | 25.26899565 |
| Observer 1 | 3 | 77 | L4/L5 | 2 | 2.037255087 | 0.277847552 |
| Observer 1 | 3 | 79 | L1/L2 | 3 | 3.236941607 | 5.821651974 |
| Observer 1 | 3 | 79 | L2/L3 | 3 | 1.422610833 | 6.336495644 |
| Observer 1 | 3 | 79 | L3/L4 | 3 | 6.118973136 | 3.215498371 |
| Observer 1 | 3 | 79 | L4/L5 | 3 | 3.910548668 | 1.12561186 |
| Observer 1 | 3 | 80 | L1/L2 | 3 | 4.528001915 | 6.609421454 |
| Observer 1 | 3 | 80 | L2/L3 | 0 |  |  |
| Observer 1 | 3 | 80 | L3/L4 | 0 |  |  |
| Observer 1 | 3 | 80 | L4/L5 | 3 | 5.891269334 | 7.709061985 |
| Observer 2 | 3 | 77 | L1/L2 | 0 |  |  |
| Observer 2 | 3 | 77 | L2/L3 | 0 |  |  |
| Observer 2 | 3 | 77 | L3/L4 | 3 | 3.736320486 | 9.341052357 |
| Observer 2 | 3 | 77 | L4/L5 | 0 |  |  |
| Observer 2 | 3 | 79 | L1/L2 | 3 | 4.862532376 | 7.564277895 |
| Observer 2 | 3 | 79 | L2/L3 | 3 | 7.685679607 | 11.20834936 |
| Observer 2 | 3 | 79 | L3/L4 | 3 | 5.416279998 | 15.05603813 |
| Observer 2 | 3 | 79 | L4/L5 | 3 | 8.645401185 | 9.779583367 |
| Observer 2 | 3 | 80 | L1/L2 | 3 | 10.48911951 | 9.829161334 |
| Observer 2 | 3 | 80 | L2/L3 | 0 |  |  |
| Observer 2 | 3 | 80 | L3/L4 | 0 |  |  |
| Observer 2 | 3 | 80 | L4/L5 | 3 | 16.9077151 | 10.96952788 |
| Observer 1 | 6 | 77 | L1/L2 | 0 |  |  |
| Observer 1 | 6 | 77 | L2/L3 | 0 |  |  |
| Observer 1 | 6 | 77 | L3/L4 | 3 | 3.806548061 | 4.780927319 |
| Observer 1 | 6 | 77 | L4/L5 | 3 | 8.674430179 | 4.972939077 |
| Observer 1 | 6 | 79 | L1/L2 | 3 | 6.826835739 | 10.34849498 |
| Observer 1 | 6 | 79 | L2/L3 | 3 | 1.099022805 | 10.456973 |
| Observer 1 | 6 | 79 | L3/L4 | 3 | 8.134468478 | 13.38464771 |
| Observer 1 | 6 | 79 | L4/L5 | 3 | 10.36477399 | 11.49756423 |
| Observer 1 | 6 | 80 | L1/L2 | 3 | 4.496356846 | 5.211896777 |
| Observer 1 | 6 | 80 | L2/L3 | 0 |  |  |
| Observer 1 | 6 | 80 | L3/L4 | 0 |  |  |
| Observer 1 | 6 | 80 | L4/L5 | 3 | 17.77478387 | 6.787473695 |
| Observer 2 | 6 | 77 | L1/L2 | 0 |  |  |
| Observer 2 | 6 | 77 | L2/L3 | 0 |  |  |
| Observer 2 | 6 | 77 | L3/L4 | 3 | 18.62739463 | 0.350178077 |
| Observer 2 | 6 | 77 | L4/L5 | 3 | 19.22927507 | 13.74632982 |
| Observer 2 | 6 | 79 | L1/L2 | 3 | 9.168807046 | 9.67038078 |
| Observer 2 | 6 | 79 | L2/L3 | 3 | 9.57436879 | 5.739636458 |
| Observer 2 | 6 | 79 | L3/L4 | 3 | 7.81893107 | 5.551935317 |
| Observer 2 | 6 | 79 | L4/L5 | 0 |  |  |
| Observer 2 | 6 | 80 | L1/L2 | 3 | 16.47804153 | 3.33985373 |
| Observer 2 | 6 | 80 | L2/L3 | 0 |  |  |
| Observer 2 | 6 | 80 | L3/L4 | 0 |  |  |
| Observer 2 | 6 | 80 | L4/L5 | 3 | 13.29483433 | 8.21598698 |
| Observer 1 | 12 | 77 | L1/L2 | 0 |  |  |
| Observer 1 | 12 | 77 | L2/L3 | 0 |  |  |
| Observer 1 | 12 | 77 | L3/L4 | 3 | 11.07839438 | 2.653488216 |
| Observer 1 | 12 | 77 | L4/L5 | 3 | 5.589713455 | 9.110588344 |
| Observer 1 | 12 | 79 | L1/L2 | 3 | 6.272341265 | 9.574116524 |
| Observer 1 | 12 | 79 | L2/L3 | 0 |  |  |
| Observer 1 | 12 | 79 | L3/L4 | 0 |  |  |
| Observer 1 | 12 | 79 | L4/L5 | 3 | 3.024304473 | 1.386734345 |
| Observer 1 | 12 | 80 | L1/L2 | 3 | 8.527320208 | 3.308618547 |
| Observer 1 | 12 | 80 | L2/L3 | 3 | 1.985884304 | 1.53981499 |
| Observer 1 | 12 | 80 | L3/L4 | 3 | 3.618388045 | 2.897743648 |
| Observer 1 | 12 | 80 | L4/L5 | 3 | 0.546767167 | 3.361252429 |
| Observer 2 | 12 | 77 | L1/L2 | 0 |  |  |
| Observer 2 | 12 | 77 | L2/L3 | 0 |  |  |
| Observer 2 | 12 | 77 | L3/L4 | 3 | 5.935501547 | 5.349206467 |
| Observer 2 | 12 | 77 | L4/L5 | 3 | 4.803829684 | 4.727150735 |
| Observer 2 | 12 | 79 | L1/L2 | 3 | 4.358119957 | 6.680780649 |
| Observer 2 | 12 | 79 | L2/L3 | 3 | 6.471480213 | 5.06683294 |
| Observer 2 | 12 | 79 | L3/L4 | 3 | 9.313559896 | 7.635951426 |
| Observer 2 | 12 | 79 | L4/L5 | 3 | 11.50936086 | 4.166709238 |
| Observer 2 | 12 | 80 | L1/L2 | 3 | 10.38567589 | 8.784041249 |
| Observer 2 | 12 | 80 | L2/L3 | 0 |  |  |
| Observer 2 | 12 | 80 | L3/L4 | 0 |  |  |
| Observer 2 | 12 | 80 | L4/L5 | 3 | 7.422314107 | 6.942841635 |
