# Supplementary material for: Feasibility of low-field magnetic resonance imaging (lf-MRI) for longitudinally evaluating experimentally induced lumbar intervertebral disc injuries in goat models (Capra hircus): A pilot study
Source: PLoS One. 2026 Feb 17;21(2):e0325577. doi: 10.1371/journal.pone.0325577 (PMC12912563; doi:10.1371/journal.pone.0325577)
Supplement: Attachment — Submitted filename: Response to Reviewer 2.docx [file pone.0325577.s009.docx]

Reviewer #2: 1) Please adjust the vertical lines position in Table 2 to improve the separation between columns, as the current format in first columns is confusing.
**The vertical lines have been adjusted to improve separation between columns containing values**

2) Thank you for the explanation about t-test. However, I would like to maintain the recommendation to replace the paired t-test with the Wilcoxon signed-rank test, for the following reasons:

a) Violation of the normality assumption
The paired t-test requires that the distribution of the paired differences be approximately normal, especially when the sample size is small.
When this assumption is not met, the t-test loses validity and may inflate the Type I error rate or reduce statistical power in unpredictable ways. In other words, the t-test is not robust under non-normality with small samples.

b) Your argument about Wilcoxon having “lower power” needs context…
Although the Wilcoxon signed-rank test may have slightly lower power under perfect normality, it is actually more powerful and more reliable when normality is violated, which is the case here.
Therefore, for the actual data Wilcoxon is better than paired t-test in terms of validity, and the “power” of the t-test is not guaranteed when its assumptions are violated.

c) Methodological rigor requires choosing the test that matches the data distribution
In scientific analysis, the appropriate test is not chosen based on theoretical power alone, but based on whether the assumptions are reasonably satisfied.
Using a parametric test despite known assumption violations compromises: statistical validity, reproducibility, and the credibility of the findings.
Thus, applying the standard paired t-test is not appropriate given the clear violation of assumptions.

**Thank you for your thorough suggestions and explanations. To address concerns with the chosen methods, we have performed additional analyses for the models run in this study to assess the adherence of the residuals to ANOVA assumptions, including employing rank transformations to demonstrate consistency with previously observed results. The methods section has been expanded for further detail.**

3. I find the findings presented in Table 3 rather unusual:

a) The intra-observer ICC for DHI (endplate-to-endplate) and the intra-observer ICC for DHI (bone-to-bone) are identical at booth baseline and week 3, to both observers.

b) The inter-observer ICC for DHI (endplate-to-endplate) is also identical to the inter-observer ICC for DHI (bone-to-bone) at all time points.

These results are highly improbable. Therefore, I recommend carefully revisiting and fully verifying the ICC analysis.

**Thank you for bringing this to our attention. The data for bone-to-bone measures were accidentally transposed into the cells for endplate-to-endplate measures. Table 3 now reflects to correct data.**
